# Of Springs

**Published:** 1869-04

**Authors:** Thos. K. Beecher


### OF SPRINGS.

BY THOS. K. BEECHER.

No, not about deceitful and hope-deferring springs, such as curse this latitude on the Atlantic side of the continent, not a word of them. Would we had never known them, or were not likely to meet another.

Nor of gushing springs that smoke on frosty mornings and keep their channels musical beneath the ice all winter long, for cattle to break into with their feet and drink their fill. Not of them.

Nor of ferocious springs, the leaps that cats make upon mice and little birds, or that tigers make upon antelopes and wild zebras, crunching vertebræ or ripping bellies. Not of such springs, no, not a word.

But of elastic springs, that "give and take," and ease up many a jar, and make life's loads ride smoothly;—these are the springs of which we think but little, while we owe them much.

Will you try an experiment, reader? Take WEBSTER'S Dictionary (or any block or stone no thicker,) and put it on the floor; then stand on it and jump off. "*Pshaw, that's nothing!*" Very well; try again, and keep your knees stiff, make your legs stake-like and springless as you jump.——There, you have had a lesson upon springs. Many a time your knee joints have saved your teeth and tongue from unnecessary and painful acquaintance.

Of course in these modern days when Anatomy and Physiology are taught to children in school, we need not enlarge upon the cartilages between the vertebræ, elastic springs or cushions set between the bones, that the head may ride, and not be jarred. In one sentence only, we allude to the elegant curves of the back bone, gracefully yielding to every sudden force. But for these curves no man could ride a trotting horse. One single step would stun him. Neither is there a straight bone in the whole body. Every bone yields a little. Every bone is a spring, and wherever two bones come together, there we find cushions and lubrication!

But of all this which is writ down in doctor's books we speak no further. Elasticity is a law of life and youth and growth. Let's look for other springs that fend off fatal joltings.

A railway engine can run a few miles, and a part of an hour, after its tank has gone dry. But there is very little margin. The engine must eat and drink, or stop. Not so with men and beasts. Meals are comfortable at their appointed times. They become desirable as hunger grows. But the soldier and sailor can, if need be, go on unfed many useful hours before he uses up his margin, or exhausts his *spring* of endurance.

Sleep is a necessity. But man is not, need not be a slave to sleep. Sleep waits upon our convenience. There is room to come and go. There is margin—*spring*. And so the Methodist keeps watch-night with prayer and pious vow, and the gay boys go to a ball, and hail New Year with mirth and music. Neither watch-night nor New Year's ball were possible but for the *spring* there is between sleepiness and sleep. But mark well, when a man has used up his margin, and taken all the life out of his spring, he MUST sleep or go crazy.

The Mississippi river meanders down its valley on a grand scale. But there is a limit to its meanderings. A line of lime-stone bluffs define the "bottom-lands," and the river can wander and vary its course from five to fifteen miles; no more. Rocks rule it when it has used up its margin. So is the elasticity and the freedom which man enjoys. He can meander, but there are bluffs of eternal rock on which he will dash himself, or to which he must conform himself. "*Springs* act through a limited space only," says the mechanical professor. Precisely.

Therefore, beware how you use up your *spring*. Beware how you waste your margin. Every law that controls man is an inexorable law, applied with a *spring* or a cushion intervening. Yonder boy smoked a cigar, and got a warning. He took poison. It did not kill him. But cigars are killing thousands, when their *spring* is gone.

Every little boy or girl may tell lies in childhood, and find no harm come of it, but contrarywise, apparent good. They escape a flogging, or perhaps win a shilling. But in the long run lies do not pay. By and bye a liar cannot lie any more; he has broken his *spring*—credit. He only who has some remaining reputation for honesty is able to lie to any advantage. Lies are no benefit to a discovered liar.

One spree does not make a confirmed drunkard. He may get badly "sprung," and yet his *spring* come back again like a bow of tempered steel. But he who goes on sprees frequently, or habitually is using up his margin—destroying his *spring*. By and bye he will go on sprees no more. The sprees will go on him. And hell has no horror more horrible than those that shall haunt him. He shall beg for eternal damnation as a relief.

And there are other *springs*--social *springs*. Among men there come to pass various organizations, such as clubs, lodges, parties, churches, &c.; and all these organizations are run by rule—constitution and by-laws. When perfectly organized they grind along with fearful vigor. They do a world of good, and a world of evil. To mitigate the evils they might do, we always find in and round every grinding organization, like a party, or lodge, or church, *springs*—graceful *springs*, gentle and generous men and women, who fend off the shocks of collision, and save many a victim from the grinding machinery. Without them, all societies, sacred and secular, would prove infernal. They may not be able to prevent, but they mitigate the grinding cruelties which all societies perpetrate when they get under motion.

Private soldiers swap coffee for tobacco across the Cumberland and the Rappahannock; they give and take the news and camp talk. General orders allow no elasticity. But human nature furnishes graces not allowed in orders.

Bigots, sectaries, priests and preachers thunder in the pulpits, or threaten from before the altars—"Come away, come away—be ye separate—have no company with heretics." Yet the victims of protestants in New England, or of Romanists in the old world have found pity, succor and escape, at the hands of the loving, who could not act as a grinding mill of persecution,—graceful mercy-*springs*.

The world's great tragedy, where a priest-led people howled for the crucifixion of JESUS, is illuminated by a NICODEMUS who defended him, the WOMAN who wept for him, the SOLDIER who believed on him, and JOSEPH, who buried him. The hardness and cruelty lets up for them, and we have hope of a race that while crucifying could furnish such confessors.

——Better stop here, or a sermon will begin—a sermon of SPRINGS, with this for a text, Blessed are the peacemakers, for they ease off many a shock and soften many a blow otherwise stunning.

---

—There are six colored men in the medical department of Harvard University.

---

—New York has 25,000 tenement houses, swarming with 500,000 human beings.

